# Deciphering the connectivity structure of biological networks using MixNet

**DOI:** 10.1186/1471-2105-10-S6-S17

**Published:** 2009-06-16

**Authors:** Franck Picard, Vincent Miele, Jean-Jacques Daudin, Ludovic Cottret, Stéphane Robin

**Affiliations:** 1 CNRS UMR 5558, Université Lyon-1, Laboratoire de Biométrie et Biologie Evolutive, 43 bd du 11 novembre 1918, F-69622, Villeurbanne, France; 2 CNRS UMR 8071, Université d'Evry, INRA UMR 1152, Laboratoire Statistique et Génome, 523, place des Terrasses, F-91000 Evry, France; 3 UMR 518 AgroParisTech/INRA, 16 rue Claude Bernard, F-75231, Paris, France

## Abstract

**Background:**

As biological networks often show complex topological features, mathematical methods are required to extract meaningful information. Clustering methods are useful in this setting, as they allow the summary of the network's topology into a small number of relevant classes. Different strategies are possible for clustering, and in this article we focus on a model-based strategy that aims at clustering nodes based on their connectivity profiles.

**Results:**

We present MixNet, the first publicly available computer software that analyzes biological networks using mixture models. We apply this method to various networks such as the *E. coli *transcriptional regulatory network, the macaque cortex network, a foodweb network and the *Buchnera aphidicola *metabolic network. This method is also compared with other approaches such as module identification or hierarchical clustering.

**Conclusion:**

We show how MixNet can be used to extract meaningful biological information, and to give a summary of the networks topology that highlights important biological features. This approach is powerful as MixNet is adaptive to the network under study, and finds structural information without any a priori on the structure that is investigated. This makes MixNet a very powerful tool to summarize and decipher the connectivity structure of biological networks.

## Background

With the increasing power of high throughput technologies and storage capacities, it is now possible to explore datasets which are in the form of complex networks. Many scientific fields are concerned by these major advances, such as physics, social sciences, and molecular biology [1,2]. One characteristics of interest when studying complex networks is their topology or the way particules, proteins or social agents interact [1]. More generally, studying the topology is crucial to understand the organization of networks, as structure often affects function. Since networks show complex structural patterns, one common task is to find an appropriate way to summarize their structure. Many indicators have been proposed for this purpose: the degree distribution [3], the clustering coefficient [2,4], and the small world property [1] are among the most popular. However since summarizing a topology using those indicators gives a crude view of the networks topology, another research direction has been to gather nodes that behave similarly from the point of view of a user defined criterion [5-7].

Clustering methods that have been proposed are mainly focused on community detection, *i.e. *they aim at finding groups of nodes that are highly intra-connected and poorly inter-connected [8]. Hierarchical versions of these methods are also available [5]. However, when performing exploratory data analysis, it may be difficult to search for a particular structure. Real networks may not show community structure for instance, or may be characterized by various connectivity patterns among which community is only one feature.

Model-based clustering is a powerful alternative to those methods, as the model underlying the algorithm allows the blind search of connectivity structure without any *a priori *[7,9,10]. The basics of this strategy is to consider that nodes are spread among an unknown number of connectivity classes which are unknown themselves. Many names have been proposed for this model, and in the following, it will be denoted by MixNet, which is equivalent to the Block Clustering model [9].

When using MixNet one central question is the estimation of the parameters, and the associated optimization method. Bayesian strategies have been proposed, but they are limited as they can handle networks with hundreds of nodes only [9]. Heuristics have also been proposed for this problem [10]. In this work, we present the MixNet software program which is the first publicly available software that fits mixture models on large networks using non Bayesian maximum likelihood estimation. The statistical developments associated with this software have been published elsewhere [7], and our algorithm uses a variational approach that has been developed in the context of graphical models [11]. Here we consider the application of MixNet to different biological networks such as regulatory, cortex, foodweb and metabolic networks. We show how flexible the method is, how it summarizes the connectivity structure of a complex network, and how this summary can be used to understand topology-based biological features.

## Results

### Brief recall of MixNet principles

In this first paragraph we briefly recall the principle of mixture models when applied to random graphs.

This is a general setting that has been developed extensively from the statistical point of view [7,9,10].

**Figure 1:**
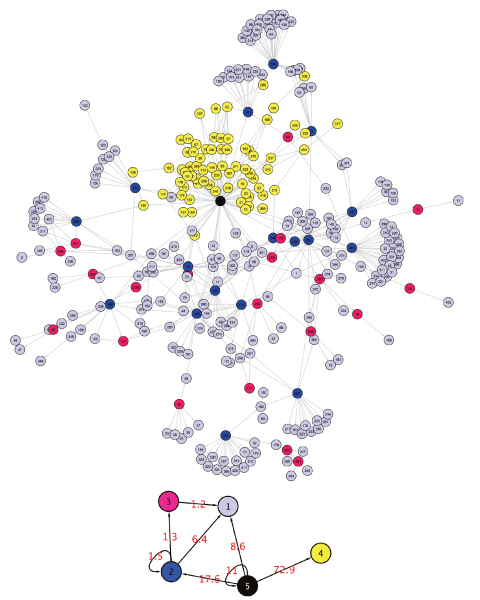
**E. Coli TRN with 5 MixNet classes with proportions**.  = 65.49,  = 5.18,  = 7.92,  = 21.10,  = 0.30

The network is modeled as a random graph with **X **representing its connectivity matrix, such that *X*_*ij *_= 1 if nodes *i *and *j *are connected and 0 otherwise. In this article, we consider directed networks, such that *X*_*ij *_may be different from *X*_*ji*_. The idea of MixNet is to consider that nodes can be spread into *Q *connectivity classes which are hidden, with *Q *being unknown as well. Then we consider that there exists a sequence of hidden label variables **Z **such that *Z*_*iq *_= 1 if node *i *belongs to class *q*. The parameters of this model are ***α***, the proportion of each group, and ***π ***the connectivity of the groups, such that *π*_*q*ℓ _represents the probability for a node of group *q *to be connected to a node from group ℓ (given in percentage in the sequel). To this extend, ***π ***is a summary of the connectivity of the original network, at the group level. MixNet results can be displayed in two ways. The first intuitive representation is to map the MixNet classes on the nodes of the network as in Figure 1. However, this view may not be informative when too many nodes/colors are present. The second way is to give a graphical representation of the connectivity matrix ***π ***which provides a synthetic view of the intensity and direction of connexions between and within MixNet classes (Fig. 1, Table 1). Then the purpose is to interpret such a summary, and our work aims at showing how biological information can be extracted from MixNet results. A classical difficulty when using clustering techniques is to determine how many clusters there are. The advantage of model-based clustering is that it gives a framework for deriving theoretical criteria for model selection. However, our point is that since there is no "true" number of clusters, it may be valuable to study the results given with different configurations. To this extend, we will use two criteria in this article. The first one is called the Integrated Classification Likelihood (ICL [7]), it is based on a penalization of the likelihood of the model. The second one is called the "adaptive strategy". Its principle is to study the increase of the likelihood according to the dimension of the model, and to select the number of clusters for which this increase is less significant [12]. These criteria are briefly described in the Method section.

**Table 1:** Connectivity matrix for E. Coli TRN with 5 classes. The probabilities of connexion are given in percentage, and probabilities lower than 1% are not displayed.

	MixNet Classes				
	1	2	3	4	5
1	.	.	.	.	.
2	6.40	1.50	1.34	.	.
3	1.21	.	.	.	.
4	.	.	.	.	.
5	8.64	17.65	.	72.87	11.01

alpha	65.49	5.18	7.92	21.10	0.30

### A meta-regulation diagram in the TRN of E. Coli

Transcriptional regulatory networks (TRN) constitute one important example of biological networks that are studied from the structural point of view. Nodes of the network correspond to operons which are linked if one operon encodes a transcription factor that directly regulates another operon. Such networks have been shown to share some important properties, such as a relative sparseness, a very low number of feed back circuits, and a hierarchical organization [13]. Thus grouping operons based on their connectivity structure appears essential to understand the wiring diagram of such complex networks. In this paragraph, we consider the connex component of the the E. Coli TRN [14].

#### Summarizing regulatory structure: the MixNet representation

The clustering results with 5 classes (given by the ICL criterion) gives a rough picture of the network's structure. The connectivity matrix *π *of the TRN is characterized by (*i*) empty rows and (*ii*) small diagonal elements (Table 1): (*i*) means that some groups are made of strictly regulated operons (nodes that receive edges only), and (*ii*) that there is no community structure, *i.e. *there is no group which is heavily intra-connected and poorly inter-connected. This result is coherent with the structure of regulatory circuits which form cascades of regulations without feedback [13], meaning that nodes do not share modularity patterns in this regulatory network. Figure 1 indicates that the majority of operons are regulated by very few nodes. At this resolution level, the network is summarized into regulated operons (groups 1 and 4), which receive edges only. These two groups are distinguished based on their regulatory elements: operons of group 4 are regulated by crp only (which makes its own group), whereas operons of group 1 are regulated by many cross-talking elements (group 2, 3, and 5).

#### Meta Motifs of regulation

It has been shown that some motifs like the popular Feed Forward Loop constituted a core structure of the E. Coli regulatory network [14]. When looking at Figure 1, it appears that MixNet exhibits the same global structures at the group level. Groups 5 and 4 form a Single Input Module (SIM), *i.e. *one TF regulating other operons that do not communicate . Similarly, groups 2-3-1 and 2-5-1 form a "meta" Feed-Forward loop. In both cases the effector group is group 1, and groups 2 and 3 can be viewed as information relays.

#### Getting a more detailed picture

**Figure 2:**
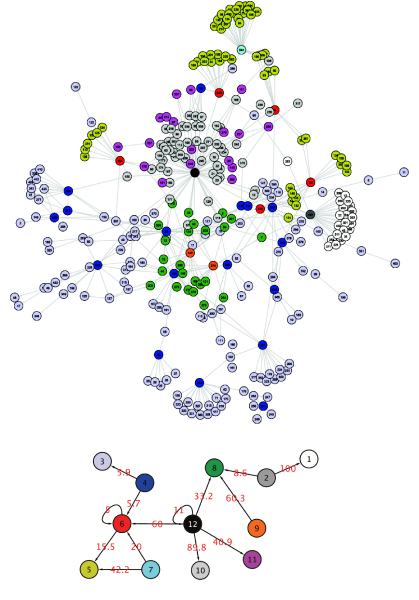
**E. Coli TRN with MixNet 12 classes with proportions**.  = 6.66,  = 0.30,  = 37.10,  = 5.35,  = 16.61,  = 1.52,  = 0.30,  = 8.59,  = 0.61,  = 16.84,  = 5.81,  = 0.30

The adaptive strategy selects 12 groups which highlight the hierarchical structure of the regulation wiring diagram (Figure 2). The majority of nodes are strictly regulated operons (groups 1, 3, 5, 8, 10), whereas regulators are clustered into small groups that are distinguished based on their connectivity patterns and on their targets. For example yhdG_fis (group 2) regulates nodes of groups 1 and 8, operons of group 9 (fnr, narL) regulate operons of group 8. MixNet can also be used to detect operons that act as global TF from the connectivity point of view. For instance, rpo operons are clustered in "regulatory" classes (operon rpoE_rseABC forms group 7 on its own). This result is not surprising though, as rpo operons are involved in the *σ *unit of the RNA polymerase. More generally, beyond groups that are made of unique major regulatory elements, MixNet gather "regulatory-like" elements together. For instance, group 4 is made of both global TF and *σ *factors (Table 2).

**Table 2:** Repartition of the E. Coli TRN in MixNet classes.

Operon	class id	out degree	in degree
yhdG_fis	2	26	0

arcA^†^	4	20	1
argR	4	6	0
cytR	4	7	0
fadR	4	5	0
FruR	4	7	0
himA^†,‡^	4	21	0
hns^†^	4	7	1
lrp^†^	4	14	0
marRAB	4	5	1
metJ	4	4	0
nlpD_rpoS*	4	14	0
ompR_envZ^†^	4	6	1
oxyR^†^	4	4	0
purR^†^	4	16	0
rob^†^	4	12	0
rpoN*	4	13	0
soxS^†^	4	6	1

cpxAR^†^	6	9	1
flhDC	6	7	3
fliAZY*	6	12	2
fur^†^	6	9	1
rpoH*	6	10	4

rpoE _rseABC*	7	24	0
fnr^†^	9	22	0
narL^†^	9	13	0

crp^†^	12	72	0

List of operons which correspond to regulatory operons in the Coli regulation network with *Q *= 12 groups. ^† ^Global TF from [35]. Note that flhDC is a master compound regulator for motility and chemostatis, and has not yet been reported to regulate other TFs [36]. ^‡^himA is the a-subunit of the Integration Host Factor. * for *σ*-factors.

Meta motifs are also present in this representation: a Meta Feed Forward Loop (5-6-7) and Single Input Modules (12-10, 12-11, 12-8, 2-8 and 2-1). Their formation is due to groups 12 and 2 which are made of one operon only (crp and yhdG_fis respectively). Another meta motif is the Dense Overlapping Regulon (DOR motif, groups 4-3). A DOR motif is formed when a set of operons are each regulated by a combination of a set of input transcription factors.

### Discovering Hub families in the macaque cortex network

The dataset consists in cortical regions connected by inter-regional pathways in the Macaque Cortex [15]. As brain function is based on inter-regional connexions, studying the way cortical regions interact may offer new perspectives in the comprehension of information flows within the brain. It appears that particular brain regions may play different roles: some regions can be at the "center" of a particular part of the network, meaning that a lot of information will pass through them, whereas other parts of the network may be more "peripherical". Consequently, identifying central zones would be important, as their lesion may compromise the integrity of the whole network.

From a topological view, finding those "hubs" as focused much attention, with a popular definition based on degree. However, there exists many ways for a node to be a hub, and degree is only one criteria. As there is no formal definition of what a hub is, there are many different hubs (provincial and central). This is why multi-criteria strategies were developed to find nodes that can be called "hubs" [15]. From a methodological point of view, this approach seems to be limited as the resuting hubs will be criteria-dependent. The gain of MixNet is that the model can be used to find those hubs. Indeed, using the underlying missing data framework, MixNet will find nodes that connect heavily to other nodes in the network, and that share this connectivity pattern (a class of hubs for instance).

#### Interpretation of MixNet results

**Figure 3:**
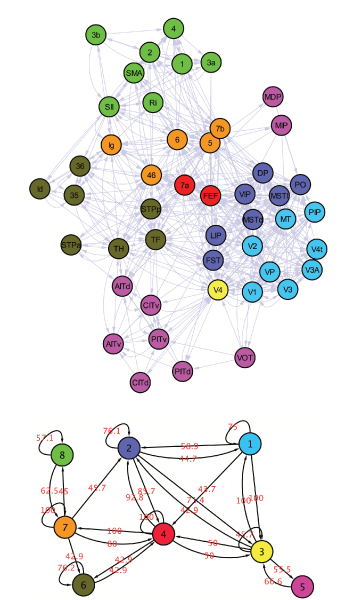
**Macaque Cortex Network with 8 MixNet classes, with proportions**.  = 17.0,  = 14.9,  = 2.1,  = 4.3,  = 19.2,  = 14.9,  = 10.6,  = 17.0



The dorsal visual stream area is a very densely connected zone in the brain, and has been viewed as homogeneous in a previous study [15]. On the contrary, MixNet emphasizes different connectivity behaviors (Figure 3). This zone is split into 3 classes (1-2-3) and MixNet still catches the strong inter-class connexion pattern . This split is explained by the intensity of connexions with other zones, and by the differences in flows direction (balanced flow for class 2, unbalanced for class 1). MixNet identifies hubs like V4, a provincial hub that constitutes a group on its own (group 3), but also sets of hubs like the Frontal Eye Field (FEF) and node 7a, that are known to receive and send many long range pathways and to connects visual and sensimotor zones respectively. Those hubs form class 4 which is also responsible of the split of the dorsal visual stream area, since inter-classes connectivity probability are very different:

Despite different functions, FEF and 7a form a class of connector hubs that allows the communication between zones that do not connect directly (classes 3-7, 3-6, 6-2, 6-1, and 1-7). This pattern is also present for class 7 that connects classes 6 and 8, with node Ig not declared as a hub based on different criteria (just below the limit [15]), whereas MixNet emphasises that its connectivity pattern is a "hub" pattern. From a histological perspective, V4 mediates information flow between two groups of areas, one belonging predominantly to the dorsal visual stream (groups 1 and 2) and the other belonging to the ventral visual (group 5, without MP and MIP). Consequently, the partition given by MixNet can also be related to geographic areas in the cortex. This can be explained by the geographic organization of the connexions within the brain. Similarly, a majority of zones of groups 6 7 and 8 belong to the parietal frontal lobe which corresponds to somatosensory and motor areas.

#### Comparison with a module identification method

**Figure 4:**
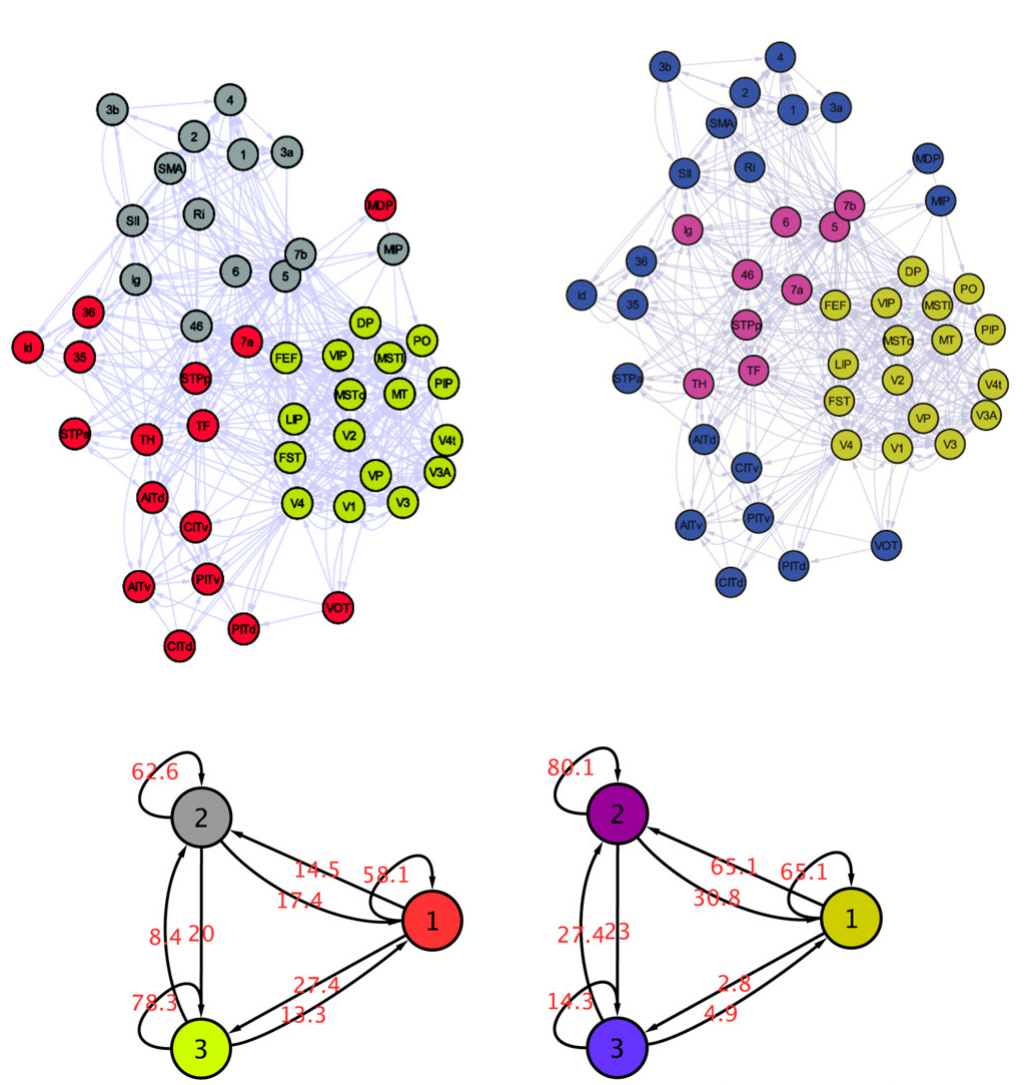
**Macaque Cortex Network Method comparison**. Left Guimera method, Right: MixNet.

Since the network of brain cortical regions is highly connected (47 nodes, 505 interactions) most cortical regions are inter-connected with different intensities. Consequently, it may be of interest to identify modules in this network. We use the detection algorithm based on simulated annealing, which aims at maximizing the modularity of a partition, and which finds the number of modules automatically [8]. This method identifies 3 modules, and we compare the partitions with a 3-class MixNet partition (Figure 4). One module is identified by both methods and corresponds to the set of cortical regions that constitute the visual stream region. The remaining 2 modules are different: while MixNet identifies a class of connector hubs (class 2 which mediates the connexions between 1 and 3), the modularity-based method identifies 2 highly intra-connected modules which belong to the ventral visual and the parietofrontal lobes. In this example, MixNet results may be more relevant from the information flow point of view, whereas the modules maybe more interesting from the histological point of view. This makes both approaches very complementary.

### Summarizing trophic relationships in food-web networks

Food webs are networks that describe the trophic links among diverse species. They provide a complex picture of species interactions and ecosystem structure. Deciphering rules that govern their formation and evolution has received much attention [16], and the study of their structure is also an active research field [5,17]. The food web under study is made of chalcid wasps from the *Tetramesa *species feeding on different grass species [18,19]. Among these wasps, some are true herbivores, many are parasitoids, and some are parasitic at early larval stages and herbivorous in later stages. The term parasitoid is used to describe the strategy in which during its development, the parasite lives in or on the body of a host. Therefore, the food web shows 5 levels of organization: plants, herbivores, parasitoids, hyperparasitoids and hyper-hyperparasitoids. Then a trophic link is considered between two insects when one insect is observed within one host, since development of parasitoid insects takes place within or on the host species. The original article points out that there is a dissymmetry among the specificity of the different trophic levels: while the lower two trophic levels (herbivores and primary parasitoids) are characterized by extreme host specificity, the top two trophic levels (hyperparasitoids and hyperhyperparasitoids) comprise more generalized omnivores.

This example has recently been used to illustrate a clustering method based on hierarchical agglomeration [17]. The provided results have the advantage of showing different degrees of precision, with the highest degree reflecting specific herbivore-parasite communities [17]. However, the hierarchy may not be present at every scale, as the network is not a tree. This is a classical criticism that can be made to hierarchical clustering in general: it will find hierarchy even if the data are not structured hierarchically. Furthermore, the hierarchical framework hampers the use of edge orientation, seeing the network as a non-directed network, whereas it is directed by definition, the orientation of the links giving the trophic relationship between organisms.

#### Summarizing trophic relationships in the wasps network

**Figure 5:**
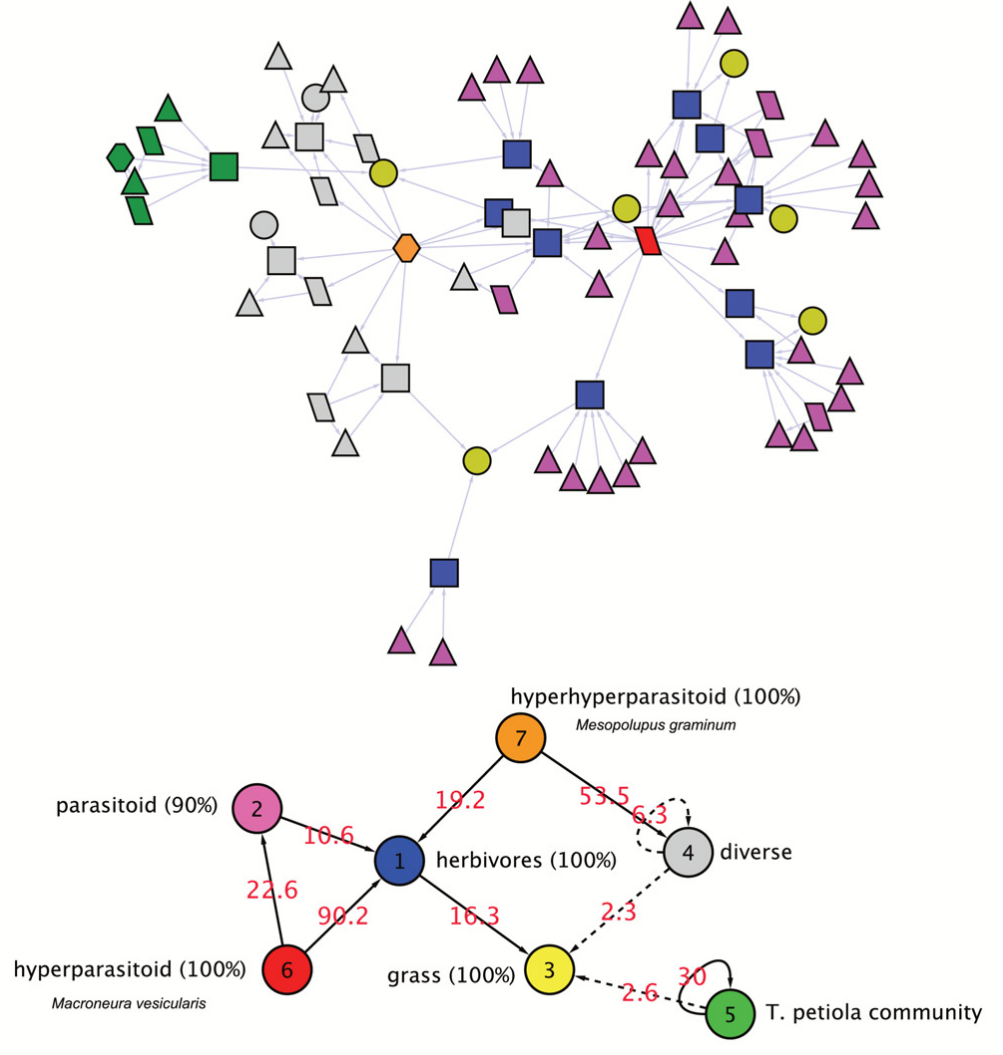
**Foodweb network with 7 MixNet classes, with proportions**.  = 14.0,  = 44.4,  = 8.6,  = 22.3,  = 8.0,  = 1.3,  = 1.3

The adaptive strategy gives 7 classes among which *Macroneura vesicularis *and *Mesopolobus graminum *constitute hubs that have different targets (Figure 5, Table 3). Then herbivores are connected to the class of grass species, and are infected by those hubs. MixNet exhibits the low specificity of hyperparasitoids, as the hub *Macroneura vesicularis *is connected to parasites as well as herbivores. This is also illustrated by the connexions of *Mesopolobus graminum *(Class 7) to herbivores (Class 1) but also to class 4 which has no specific pattern in terms of trophic levels. Actually *Mesopolobus graminum *creates a partitioning of the network, since cluster 4 is formed by nodes that connects together or with the hub, but not with other parts of the network. More than hub identification, MixNet can also identify local hierarchies. For instance class 5 is made of a community centered around the herbivore *Tetramesa petiolata*. This illustrates a case of narrow host range which is typical of communities centered on herbaceous plants [18].

**Table 3:** Repartition of trophic levels among MixNet Classes for the foodweb network.

	MixNet Classes							Mean Degree	
	1	2	3	4	5	6	7	In	Out
grass	0	0	6	2	0	0	0	2.00	0.00
herbivore	10	0	0	4	1	0	0	4.53	1.06
parasitoid	0	30	0	7	2	0	0	0.64	1.05
hyperparasitoid	0	4	0	4	2	1	0	0.36	3.82
hyperhyperparasitoid	0	0	0	0	1	0	1	0.00	7.00

Mean In degree	4.90	0.41	2.33	1.59	1.50	0	0		
Mean Out degree	1.10	1.29	0	1.17	1.66	17	11		

### Summarizing Reaction interplay in metabolic networks

Metabolic networks constitute a major instance of biological networks, whose comprehension appears crucial in the understanding of the functionning of the cell. In this example, we consider the metabolic network of the gamma-proteobacterium *Buchnera aphidicola*. This bacterium is an endosymbiont which lives inside some specialised cells of the aphids. An intimate association exists between these two organisms since one can not live without the other [20]. The symbiosis is nutritional: each organism provides metabolites that the other can not synthesize. Nutritional analyses showed that the essential role of *B. aphidicola *in the symbiosis is to supply essential amino acids that the aphid can not produce. The very long association between these two organisms (over 150 millions years) and the strictly vertical transmission of the endosymbiont induced a drastic reduction of its genome, affecting its metabolic capacities but preserving especially the symbiotic functions [21].

In this example, the data is a directed network with reactions as nodes which are connected if one reaction produces the substrate of the other. One reaction has been declared as being irreversible if it appears always in the same direction in MetaCyc [22], whatever the metabolic pathway. This strategy requires an additional filtering step that accounts for some compounds which would create numerous connections that might not be biologically meaningful. Indeed, some metabolites as ATP and NADP often act as co-factors in reactions but do not transfer matter to the main substrates. Not dealing with these metabolites induces false topologies in the metabolic networks and thus wrong biological deductions [23]. Since MixNet aims at finding structure, this kind of non informative structure hampers the discovery of smaller-scale structures (data not shown). In order to avoid artefactual structures due to cofactors partipations, we used a filter to remove substrat-products couples corresponding to cofactors [24]. Subproducts such as phosphate from the tandem ATP-ADP, and H_2_O have also been removed.

**Figure 6:**
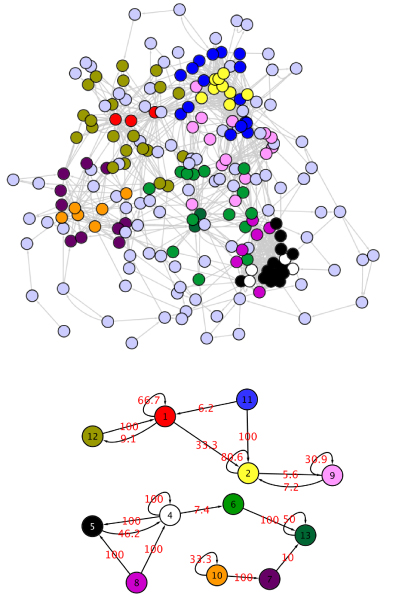
**Metabolic network with 13 MixNet classes with proportions**.  = 1.4,  = 4.1,  = 45.4,  = 1.8,  = 6.0,  = 6.3,  = 4.6,  = 2.8,  = 6.4,  = 2.8,  = 7.4,  = 10.1,  = 0.9.

The first result of MixNet is that 45% of the reactions of the metabolic network of *B. aphidicola *are "chain-like" reactions that are not sufficiently structured from the connectivity point of view to be split into more subsets of reactions. Indeed, Class 3 has a mean degree close to 2 which indicates chains of reactions with only few branch lines (Table 4). It seems to be consistent with the fact that most of the redundant metabolic pathways disappeared from the metabolic network of *B. aphidicola *[21]. The twelve remaining classes form 2 meta components whose links are very loose (they are not represented on the summary plot of Figure 6, but these components are connected through reactions of class 3).

**Table 4:** Average degree for the metabolic network with 13 MixNet classes.

Mixnet Class	alpha	Ave. In Deg	Ave. Out Deg
1	1.4	24.33	7
2	4.1	24.88	8.88
3	45.4	1.52	1.40
4	1.8	16	17.67
5	6.0	10.33	2.58
6	6.3	2.78	3.14
7	4.6	6.80	1.40
8	2.8	1.16	19.67
9	6.4	5.14	6.29
10	2.8	2.66	12.33
11	7.4	1.81	10.56
12	10.1	1.54	4
13	0.9	18.50	3.50

#### Deciphering elements that structure the network

MixNet reveals two key characteristics that structure the network: compounds (phosphate, CO_2_, protons, sugars, glutamate, Isoleucine, Leucine and Valine) and the reversibility of reactions. Producer reactions are distinguished from consumers. For instance, diphosphate is produced irreversibly by reactions of class 12 and is used as a substrate to produce phosphate by class 1, which is also produced by all reactions of class 11. The distinction between producers and consumers can also be seen with the average in/out degree of each class (Table 4). It is important to note that the presence of phosphate here is not the subproduct of the transformation of ATP in ADP or other cofactor transformation. Interestingly in *B. aphidicola*, the use of phosphate as substrate occur in degradation of purines whose the products may lead to the synthesis of several other important metabolites as the chorismate, key compound in the synthesis of amino acids. A similar pattern can be found with CO_2 _that is used by reactions used by reactions of class 13, and with reactions that use/produce protons (Table 5). If we go to further details, sugars also structure the network (class 9), with reactions are span among the pentose phosphate pathway and the glycolysis.

**Table 5:** Reactions of the Buchnera metabolic network that involve protons.

MixNet class 7		
substrate(s)		product(s)
proton+cpd-602	→	cpd-1086
proton+super-oxide	→	hydrogen-peroxide+oxygen-molecule
proton+hydroxy-methyl-butenyl-dip	→	delta(3)-isopentenyl-pp
proton+hydroxy-methyl-butenyl-dip	→	cpd-4211
proton+3-dehydro-shikimate	→	shikimate
proton+2,3-dihydrodipicolinate	→	delta1-piperideine-2-6-dicarboxylate
proton+2-amino-3-oxo-4-phosphonooxybutyrate	→	1-amino-propan-2-one-3-phosphate+carbon-dioxide
proton+2-aceto-lactate	→	dioh-isovalerate
proton+methylene-thf	→	5-methyl-thf
proton+l-aspartate-semialdehyde	→	homo-ser

MixNet class 10		
substrate(s)		product(s)

erythrose-4p	→	proton+erythronate-4p
2-d-threo-hydroxy-3-carboxy-isocaproate	→	proton+cpd-7100
cpd-296	↔	proton+lipoic-acid
proton+oxygen-molecule	↔	proton
sirohydrochlorin+fe+2	→	proton+siroheme
glc-6-p	→	proton+d-6-p-glucono-delta-lactone

In component 2, we observe a very strong structure which is due to the use of glutamate, with irreversible reactions that produce glutamate from glutamine (class 8), and reactions that use glutamate (classes 5 and 4). Interestingly these consumer reactions are split because of their different reversibility despite their strong probability of connexion . Reactions of class 4 are all reversible and are involved in the metabolism of 3 Amino Acids (Isoleucine, Leucine and Valine) with a common EC number (2.6.1.42), whereas reactions of class 5 are strictly irreversible (83% of which being with EC numbers 2.6.1 and 6.3.2). The glutamate is a key compound in the synthesis of amino acids and thus plays a very important role in the symbiotic function of *B. aphidicola*. Consequently, MixNet enables to emphasize the the central role of the glutamate in the network.

## Discussion and conclusion

In this work we show how MixNet can be used to study biological network by providing an accurate summary of the main topological features that structure the network. We explored networks that show very diverse structures: the transcription and the foodweb networks are sparse and globablly structured by hubs, whereas the cortex and the metabolic network are dense with some hubs and some strongly connected components. Interestingly MixNet is adaptive to each structure, and catches very diverse features like hubs, hub families, connecting classes, cliques, and local hierarchies. This makes this tool very flexible, and very powerful to detect many features within the same network, whereas oriented clustering techniques like module identification will search for specific features only, even if these features are not in the network. Overall, the graphical representation of a network is a challenging task, and MixNet provides a global view of the network and emphasizes the key elements that make the topology. Summarizing nodes into a small number of meta-nodes linked by meta-edges gives a representation that constitutes a clear synthesis of the network topology.

Here we presented how MixNet parameters can reveal interesting features from the biological point of view. This emphasizes that MixNet is not only a computer software, but also a powerful model that can be used to simulate networks, or as a reference model under which theoretical statistics can be derived. This approach has already been demonstrated in network motifs analysis [25].

Note that the topology of a network is only one structural information that can be used to understand networks functions. It is worth being noted that the incorporation of edge direction improves the interpretability of the results, as the topology itself only constitutes a crude information. Moreover, many networks also have informations on edges: transcription regulatory networks have labeled edges (Activator/Repressor), and metabolic network have stœchiometry which reflects compounds flow in the network. A future research direction will be to use this additional information [26].

## Methods

### Data description

The transcription regulatory network has been downloaded from U. Alon web page [27]. We use only the connex component of the 1.1 version of the network, which is made of 328 nodes with 456 interactions. The food web network has been provided by A. Clauset, and is made of 86 nodes and 113 edges. The cortex network is made of 47 nodes and 505 interactions. It is available in the supplementary material of [15]. The metabolic network was build by the pathway-tools software [28] from the genomic annotations provided by the MAGE annotation platform [29]. The genome of *B. aphidicola *is quite well annotated since it can be considered as a subset of the intensively curated genome of *Escherichia coli*. Consequently, the construction of the *B. aphidicola *metabolic network is supposed to be meaningful from the biological point of view. Overall the network is made of one connex component with 946 edges and 218 nodes.

### Model Selection

In this paragraph we explain briefly the model selection procedure employed to select the number of clusters. The first criterion ICL is a particular penalized likelihood criterion: it is used to make a trade-off between a reasonable number of parameters and a good quality of fit of the data. In addition to the traditional BIC, ICL also considers the quality of the partition, meaning that it will select a number of clusters for which the classes are well separated (with low entropy). Consequently, ICL is based on the penalization of the complete-data log likelihood of the model, that accounts for the observed **X **and the missing data **Z**. The number of classes is selected such that:



with pen(*Q*) a penalty that depends on the number of nodes in the network, as well as on the number of parameters in the model [7].

The second method we employ is based on the geometrical behavior of the incomplete-data likelihood when the number of classes increases. It is an adaptive method that has been successfully employed in diverse contexts [12,30,31]. The principle of this method is to calculate the second derivative of the likelihood, and to select the number of classes for which this derivative exceeds a threshold, which is set to 0.5 in practice. This method is close to the L-curve method [12].

### The MixNet software

All the presented algorithms are implemented into the MixNet software package which is written in ANSI C++ and includes Fortran 77 subroutines from the ARPACK [32] library. Optionnal post-treatment programs written in *Perl *are also included in the package. Compilation and installation are compliant with the GNU standard procedure. The library is freely available on the MixNet webpage [33]. Online documentation and man pages are also available. MixNet is licensed under the GNU [34] General Public License.

The complexity of the algorithm is proportional to the number of edges of the network (sparse storage format), and (*n*^2^*Q*^2^) in time (where *n *stands for the number of nodes and *Q *for the number of clusters). If MixNet is run for 1 to *Q *clusters, the overall complexity is then (*n*^2^*Q*^3^). We present the speeds of execution of MixNet on the webpage [33]. Our experience is that on the studied networks, the execution speeds were similar to the simulated annealing method.

## Competing interests

The authors declare that they have no competing interests.

## Authors' contributions

FP wrote the manuscript and conducted the analysis, VM developed the MixNet software program, LC created and did the analysis of the metabolic network, JJD and SR supervised the study.

## Fundings

L. Cottret was sponsored by the ANR REGLIS Project NT05-3_45205.
